# Comparison of four different screw configurations for the fixation of Fulkerson osteotomy: a finite element analysis

**DOI:** 10.1186/s10195-023-00714-6

**Published:** 2023-06-26

**Authors:** Faruk Aykanat, Ozkan Kose, Bulent Guneri, H. Kursat Celik, Albert Cakar, Ersin Tasatan, Mihaela-Elena Ulmeanu

**Affiliations:** 1 Vocational School of Health Services, SANKO University, Gaziantep, Turkey; 2 Department of Orthopedics and Traumatology, Antalya Training and Research Hospital, Varlık mah., Kazım Karabekir cd., Muratpasa, 07100 Antalya, Turkey; 3 Department of Orthopedics and Traumatology, Adana City Education and Research Hospital, Adana, Turkey; 4 Agricultural Faculty, Department of Agricultural Machinery and Technology Engineering, Akdeniz University, Antalya, Turkey; 5 Department of Orthopedics and Traumatology, Istanbul Training and Research Hospital, Istanbul, Turkey; 6 Department of Orthopedics and Traumatology, Prof. Dr. Cemil Tascioglu City Hospital, Istanbul, Turkey; 7 Department of Manufacturing, Polytechnic University of Bucharest, Bucharest, Romania

**Keywords:** Finite element analysis, Tibial tubercle osteotomy, Fulkerson osteotomy, Screw fixation, Biomechanics

## Abstract

**Background:**

Conventionally, two 4.5 mm cortical screws inserted toward the posterior tibial cortex are usually advocated for the fixation of Fulkerson osteotomy. This finite element analysis aimed to compare the biomechanical behavior of four different screw configurations to fix the Fulkerson osteotomy.

**Materials and methods:**

Fulkerson osteotomy was modeled using computerized tomography (CT) data of a patient with patellofemoral instability and fixed with four different screw configurations using two 4.5 mm cortical screws in the axial plane. The configurations were as follows: (1) two screws perpendicular to the osteotomy plane, (2) two screws perpendicular to the posterior cortex of the tibia, (3) the upper screw perpendicular to the osteotomy plane, but the lower screw is perpendicular to the posterior cortex of the tibia, and (4) the reverse position of the screw configuration in the third scenario. Gap formation, sliding, displacement, frictional stress, and deformation of the components were calculated and reported.

**Results:**

The osteotomy fragment moved superiorly after loading the models with 1654 N patellar tendon traction force. Since the proximal cut is sloped (bevel-cut osteotomy), the osteotomy fragment slid and rested on the upper tibial surface. Afterward, the upper surface of the osteotomy fragment acted as a fulcrum, and the distal part of the fragment began to separate from the tibia while the screws resisted the displacement. The resultant total displacement was 0.319 mm, 0.307 mm, 0.333 mm, and 0.245 mm from the first scenario to the fourth scenario, respectively. The minimum displacement was detected in the fourth scenario (upper screw perpendicular to the osteotomy plane and lower screw perpendicular to the posterior tibial cortex). Maximum frictional stress and maximum pressure between components on both surfaces were highest in the first scenario (both screws perpendicular to the osteotomy plane).

**Conclusions:**

A divergent screw configuration in which the upper screw is inserted perpendicular to the osteotomy plane and the lower screw is inserted perpendicular to the posterior tibial cortex might be a better option for the fixation of Fulkerson osteotomy.

*Level of evidence* Level V, mechanism-based reasoning.

## Introduction

Tibial tubercle (TT) anteromedialization osteotomy, also called Fulkerson osteotomy, has become a standard surgical procedure in patients with patellofemoral (PF) instability associated with increased TT lateralization [1, 2]. This procedure effectively decreases the tibial tubercle–trochlear groove (TT–TG) distance and corrects the excessive Q angle [2, 3]. Secondly, it provides TT anteriorization that reduces the PF contact pressures [2]. Many previous studies have reported that Fulkerson osteotomy is a safe and effective procedure; however, it is not without complications [1–5]. Nonunion or delayed union, tibial fracture at the distal edge of the osteotomy, loss of knee range of motion, skin irritation due to prominent hardware, superficial and deep infection, failure of fixation, and neurovascular injuries have all been reported [2–5].

**Fig. 1:**
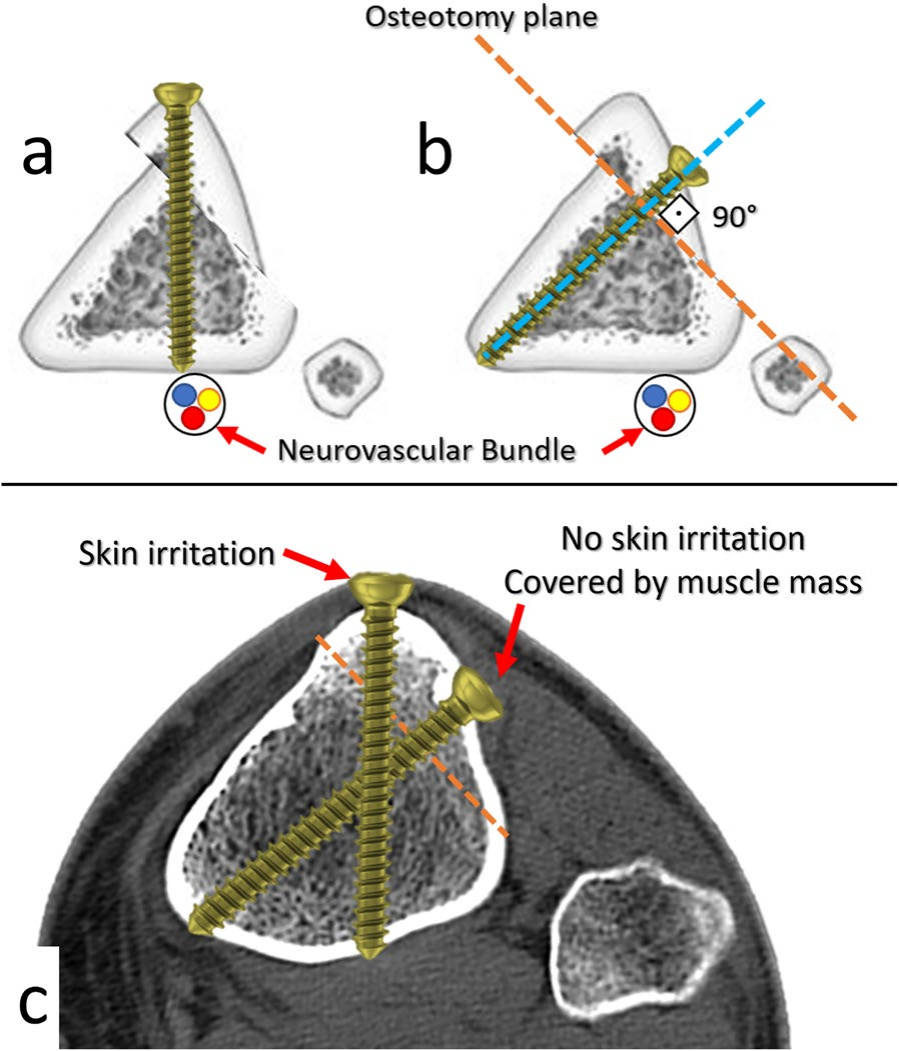
Illustration showing the insertion of screw perpendicular to the posterior tibial cortex (**a**), and perpendicular to the osteotomy plane (**b**). Please note the risk of neurovascular injury with the first configuration. A screw inserted perpendicular to the osteotomy plane is covered by the muscle mass (c)

Proper planning and performance of the osteotomy, secure fixation, and maintaining the stable fixation until the bony union is crucial to prevent implant failure and complications [3, 5]. The fixation should be stable enough for early weight-bearing and active rehabilitation to preserve knee movements and thigh muscle mass. Conventionally, two parallel 4.5 mm cortical screws inserted toward the posterior tibial cortex are usually advocated for the fixation of the Fulkerson osteotomy [6]. However, the screws inserted by this method may not provide sufficient stability since the osteotomy plane is oblique. According to the AO principles, screws placed perpendicular to the fracture or osteotomy plane provide optimal compression and reduction [7]. Furthermore, bicortical fixation is well known to be stronger than unicortical fixation [7]. But, the penetration of the posterior cortex might cause iatrogenic neurovascular injuries because the popliteal nerve and artery lie posterior to the posterior tibial cortex [8, 9]. Finally, the screw heads may remain prominent when inserted perpendicular to the posterior cortex. On the other hand, screws inserted perpendicular to the osteotomy plane might be advantageous since they move away from the direct contact area during kneeling and are covered by the muscle mass (Fig. 1).

John Fulkerson first described anteromedialization tibial tubercle osteotomy in 1983 [10]. In his original technical description, it was reported that osteotomy fixation could be done via bicortical fixation with a single cortical lag screw or unicortical with a single cancellous lag screw without penetration of the posterior tibial cortex to avoid neurovascular structures. Since then, a limited amount of biomechanical research on how to fix a tibial tubercle osteotomy has been performed in the current literature (Table 1) [6, 11–19]. Screws of various thicknesses (3.5, 4.0, 4.5 mm), numbers (two or three), designs (fully threaded or partially threaded), materials (stainless steel, titanium, and bioabsorbable polymers), cerclage applications, plate fixation, and augmentation techniques have been reported. The screws used in almost all of these studies were placed in the anterior–posterior direction, as recommended by the original description of the technique. Only three studies examine the effect of different screw configurations on the fixation stability of tibial tubercle osteotomies. Chang et al. evaluated the gap formation, contact pressure, and stress distribution on the osteotomy plane created by six different combinations by changing the configurations of the screws in the sagittal plane. In the other study, Fulkerson osteotomy with distalization was modeled, and the screws were inserted toward the posteromedial cortex in the axial plane. However, no previous study examined the effect of axial plane divergence of screws in the axial plane on the stability of standard Fulkerson osteotomy.

**Table 1:** Previous biomechanical studies on the biomechanical behavior of the various fixation methods of tibial tubercle osteotomy

Author	Year	Type of study	Model	Fixation methods	Results
Cosgarea et al.	1999	Cadaver (13 pairs)	Fulkerson osteotomy flat osteotomy	Two 3.5 mm screws (screw material unspecified)	The flat osteotomy resulted in a higher load to failure compared with Fulkerson osteotomy (1639 N versus 1166 N, *P* < 0.05)
Davis et al.	2000	Cadaver (36 unpaired knees)	Bevel-cut and step-cut flat osteotomies	Two 4.5 mm cortical screws versus 18-gauge stainless (either three- or four-level) cerclage	The failure load for the bevel-cut osteotomies repaired with two screws was 1654 ± 359 N; for the bevel-cut osteotomies repaired with three cerclage wires, 622 ± 283 N; for the step-cut osteotomies repaired with three cerclage wires, 984 ± 441 N; and for the step-cut osteotomy repaired with four cerclage wires, 1099 ± 632 N
Caldwell et al.	2004	Cadaver (40 unpaired tibia)	Flat osteotomy	Two 4.5 mm bicortical screws 18-gauge stainless four-level cerclage. Traction force at 0° and 25°	Screw constructs failed at 1429 ± 348 N (0°) and at 1925 ± 982 N (25°). Wire constructs failed at 1072 ± 260 N (0°) and at 893 ± 293 N (25°)
Warner et al.	2013	Cadaver (5 pairs)	Fulkerson osteotomy	Two 4.5 mm screws versus Three 3.5 mm screws	The maximum failure load for osteotomies secured with two 4.5-mm screws was 1459 ± 540 N, and for three 3.5-mm screws, it was 1360 ± 707 N (*P* = 0.723)
Nurmi et al.	2017	Cadaver (22 pairs)	Flat osteotomy (straight cut)	4.5-mm PLLA screws versus 4.5 mm stainless steel screws	The mean yield load was 566 ± 234 N in the bioabsorbable screw group and 984 ± 630 N in the metal screw group (*P* = 0.002)
Chang et al.	2019	FEA	Flat osteotomy	Two titanium 4.5 mm screws with six configurations: parallel horizontal screws placed at a 20 mm interval, parallel horizontal screws placed at a 30 mm interval, parallel upward screws, parallel downward screws, trapezoid screws, and divergent screws	The configuration of two parallel downward screws yielded the highest stability with the lowest fragment displacement and gap opening. The configuration of two upward screws resulted in the highest fragment displacement and gap deformation between the fragment and tibia. The stress of the osteotomized bone fragment was highest with the configuration of two upward screws
Chang et al.	2019	FEA	Flat osteotomy with three fragment shapes: step cut, bevel cut, and straight cut	Two titanium 4.5 mm screws with three configurations: parallel horizontal screws with an interval of 20 mm, trapezoidal screws with an angle of 45, and parallel downward screws with an interval of 15 mm	The step cut resulted in higher stability than the bevel and straight cut, but the stress was higher. Among the screw configurations, two parallel downward screws resulted in the highest stability, given the same fragment shape. In the horizontal configuration, the step-cut tibia developed the largest contact force to achieve stability of the bone fragment under loading
Chen et al.	2019	FEA	Flat osteotomy (step cut) with 1 mm gap formation either in proximal or distal contact surfaces	Two titanium 4.5 mm screws with six configurations: parallel horizontal screws placed at a 20 mm interval, parallel horizontal screws placed at a 30 mm interval, parallel upward screws, parallel downward screws, trapezoid screws, and divergent screws	Proximal gap model resulted inferior results compared with distal gap model in all screw configurations Among the screw configurations, two parallel downward screws resulted in the highest stability in both models
Guneri et al.	2021	FEA	Fulkerson osteotomy + distalization	Two 3.5 mm cortical screws, Two 4.5 mm cortical screws Three 3.5 mm cortical screws Three 4.5 mm cortical screws Three 3.5 mm screws with 1/3 tubular plate Four 3.5 mm screws with 1/3 tubular plate	Maximum sliding (0.660 mm), gap formation (0.661 mm), and displacement (1.267 mm) were seen with two 3.5 mm screw fixation, followed by two 4.5 mm screws, three 3.5 mm screws, and three 4.5 mm screws, respectively, in the screw group. The minimum displacement was observed with a plate, and two 3.5 mm screw fixation models
Frame et al.	2021	Cadaver (5 pairs)	Flat osteotomy	Two parallel 4.0 mm partially threaded cannulated screws versus Two parallel 4.0-mm partially threaded cannulated screws plus a nonabsorbable suture tape (FiberTape) in a figure-of-8 construct	Two specimens of the standard group exhibited clinical failure during cyclic loading to 400 N. All other specimens survived cyclic loading to 800 N The mean ultimate failure load after the pull-to-failure test was 2475 ± 554 N for the augmented group and 1475 ± 280 N for the standard group
Current study	2022	FEA	Fulkerson osteotomy	Two 4.5 mm cortical screws were tested in four different configurations (1) Both screws are perpendicular to the osteotomy plane (2) Upper screw is perpendicular to the tibial cortex; the lower screw is perpendicular to the osteotomy plane (3) Both screws are perpendicular to the posterior tibial cortex (4) Upper screw is perpendicular to the osteotomy plane; the lower screw is perpendicular to the posterior tibial cortex	The minimum total displacement was observed in the fourth scenario, which resulted in the highest stiffness

Our study hypothesized that stability would increase if the screws were placed perpendicular to the osteotomy plane. Stable fixation is essential for the early initiation of rehabilitation and weight-bearing after the osteotomy, as well as the prevention of complications.

## Materials and methods

### Study design and finite element method

This study utilized finite element methods that were carried out under static loading conditions and homogeneous isotropic linear elastic model assumptions. Besides, nonlinear contact behavior between related components was considered. Fulkerson osteotomy was generated, and the model was fixed with four different screw configurations.

FEA is a numerical method that utilizes models and simulations to examine the mechanical behavior of an object under certain physical conditions in a virtual computer environment. In the finite element method, complex 3D geometric objects are divided into small elements connected with nodes; this procedure is also called spatial discretization. Models can be created using computerized tomography data to simulate real bony geometry. Material properties, contact definitions, and boundary conditions are defined and entered into the software. Finally, force–displacement equations of each small element can easily be computed and combined for the entire structure. Thus, displacements, including sliding and gapping, and strains and stresses arising from these displacements, can be calculated and visualized throughout the model and its components.

Currently, the gold standard for assessing the initial stability of fracture or osteotomy fixation remains biomechanical testing using cadaveric models. However, FEA has several advantages over conventional biomechanical experiments. First, this method is cheaper than using fresh cadavers. Second, a complete inspection of the model throughout the entire structure is possible. Conversely, assessing internal strain and damage is exceptionally challenging in cadaver models.

Additionally, it is unaffected by geometric variations and bone quality differences between cadavers, which is a significant confounding factor. Another advantage of FEA simulations over biomechanical testing is the ability to evaluate and compare alternative implant designs or configurations of the same implant within the same bone. Moreover, new implant designs might be tested and modified without manufacturing the final prototype. Besides these advantages, FEA inherits certain disadvantages. Simplifying complex geometry and using predefined assumptions regarding material properties and boundary conditions are the significant limitations of the technique. Secondly, the divergence of the models from clinical reality, model verification, and validation are other significant obstacles that might result in misleading interpretations. Since FEA is fundamentally a metamathematical method that finds approximated solutions to biomechanical problems, errors in FEA are inevitable. Thus above-mentioned pros and cons should be considered when evaluating the outputs [20–22].

### Modeling of the Fulkerson osteotomy

Computerized tomography (CT) data of a 20-year-old male patient (height 174 cm and weight 76 kg) with recurrent patellar dislocation was used to create the tibial model. The tibial tubercle–trochlear groove (TT–TG) distance was abnormal (23 mm), and Fulkerson osteotomy was indicated for the patient. A TT–TG distance larger than 20 mm is accepted as abnormal.

The CT imaging was performed using the CT scanner (Siemens go.Up, Siemens, Munich, Germany) installed in the authors’ university hospital. The following were the scan parameters: a total of 232 axial slices were taken at 120 kV, 30 mA, slice distance of 1.0 mm, FoV: 218 mm, from the supracondylar femur to the proximal tibia. The patient gave written informed agreement to the anonymous use of the imaging files. To model and simulate the FEA scenarios, Materialise Mimics–Medical 3D image-based engineering software (Materialise NV, Belgium), SolidWorks parametric solid modeling software (Dassault Systems SolidWorks Corp, Waltham, USA), and ANSYS Workbench FEA code (ANSYS, Ltd., Canonsburg, PA, USA) were used.

**Fig. 2:**
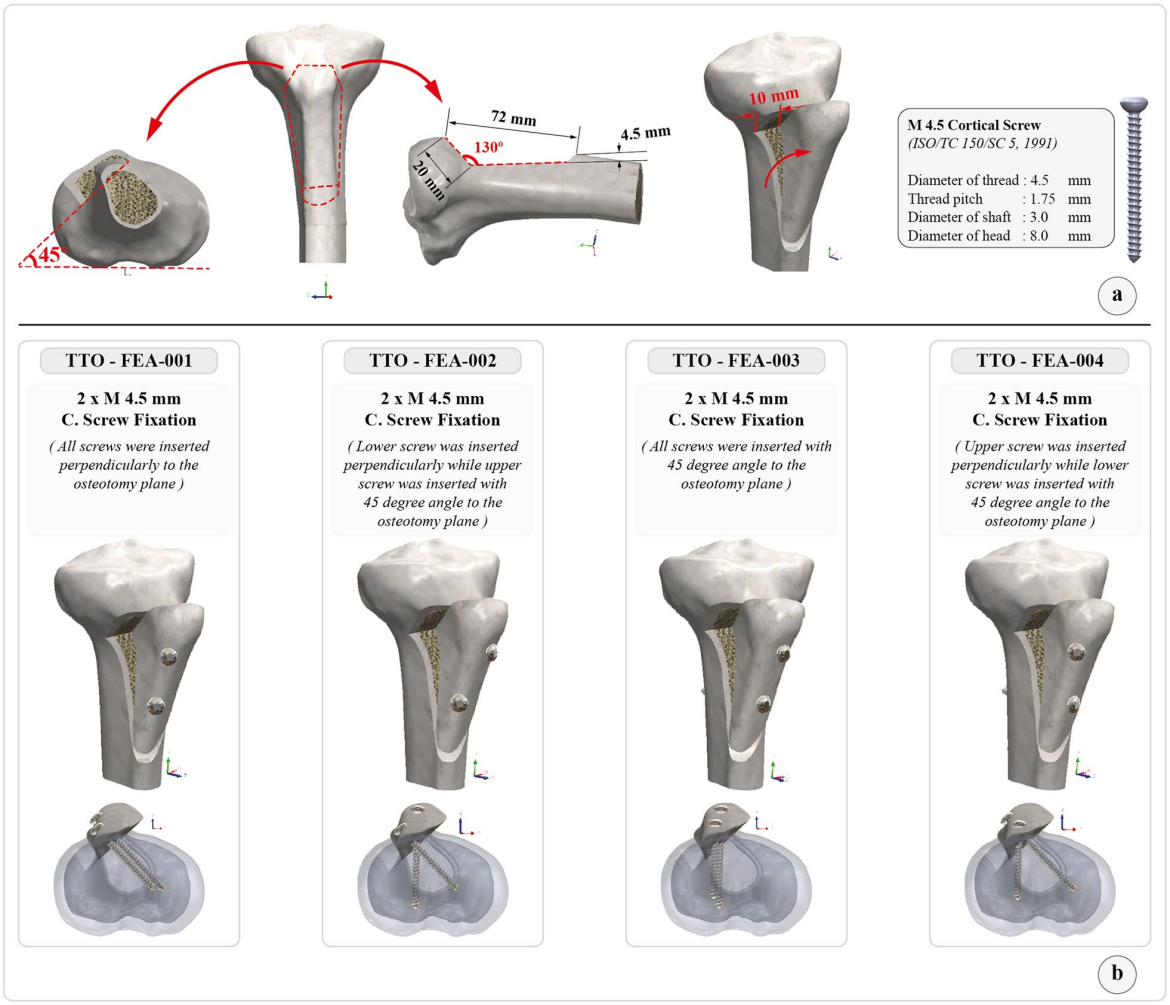
**a** Solid model showing the details of Fulkerson osteotomy. The tibial tubercle fragment is 72 mm in length, the osteotomy plane is 45° to the posterior condylar axis, and 10 mm of medialization is performed. **(b)** The screw configurations and fixation scenarios tested in FEA

The Fulkerson osteotomy was modeled following prior descriptions [10]. The osteotomy length was 72 mm, and the osteotomy plane was 45° relative to the posterior condylar axis of the tibia. Both the proximal and distal cuts of the osteotomy were slopped. A 10 mm medialization was performed. The model was fixed using 4.5 mm screws manufactured of Ti–6Al–4 V (Ti G5) alloy (Fig. 2a). There was no gap between the fragments at the osteotomy plane. For the fixation of the Fulkerson osteotomy, two parallel 4.5 mm cortical screws inserted in the sagittal plane toward the posterior tibial cortex are commonly recommended [6]. But, in line with the hypothesis in the current study (to stay away from the posterior neurovascular structures, to achieve a bicortical fixation, to capture the thicker posteromedial cortex of the tibia, and, most importantly, to achieve a fixation perpendicular to the osteotomy plane) the configuration of the screws was shifted toward the posteromedial cortex of the tibia in the axial plane. We also hypothesized that divergent screw placement might be more resistant to fragment sliding and gap formation. Thus, four different fixation scenarios were created on the basis of the above-mentioned principles (Fig. 2b).

### Boundary conditions and material properties

**Fig. 3:**
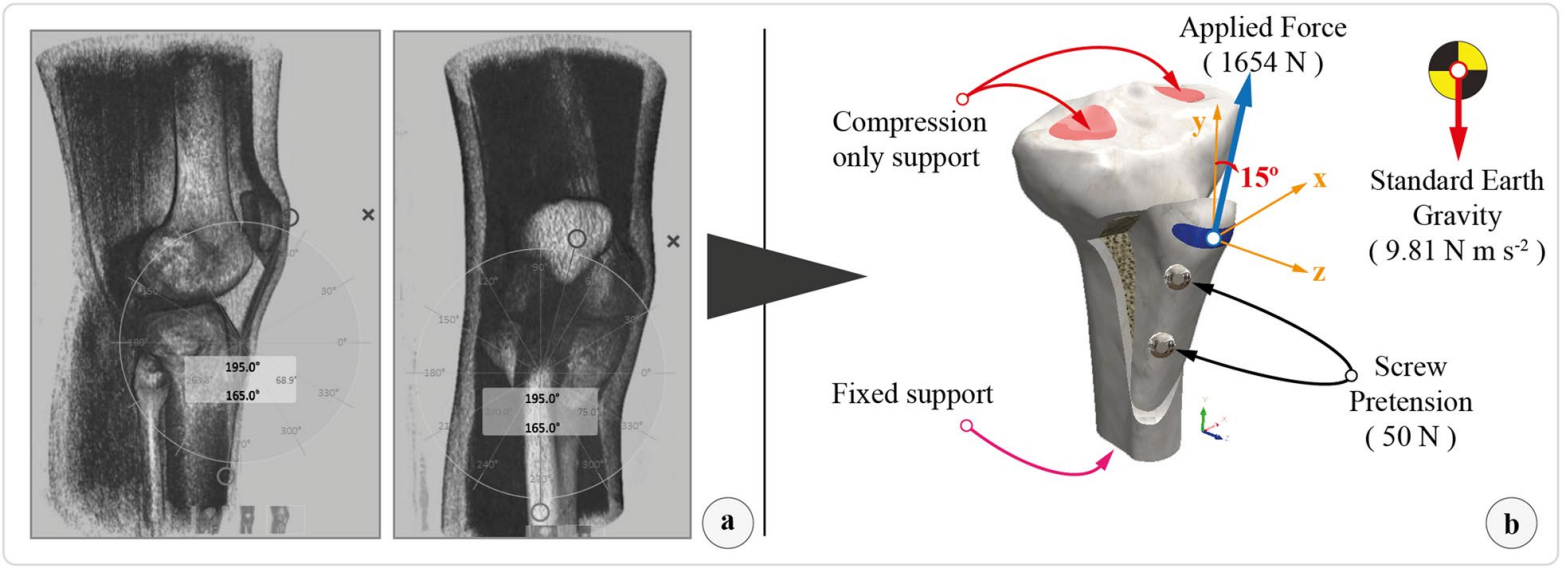
**a** Volume-rendered 3D CT image from the lateral and anterior view. The patellar tendon footprint and the patellar tendon force vector were calculated. **b** Boundary condition of the models during loading

Davis et al. [12] found that a TT osteotomy failure load fixed with two 4.5 mm cortical screws was 1654 N in a biomechanical study performed on fresh frozen cadavers. To simulate a worst-case scenario, a 1654 N traction force was applied to the patellar tendon footprint at the TT [12, 15, 16]. The total area of the patellar tendon footprint and the angle of the force vector was calculated from the CT data (Fig. 3). The tibia was fixed to the ground from its distal part in an anatomical position, and two supports at the medial and lateral condyles were placed on the proximal tibia. Frictional contact (nonlinear contact) between screw–bone surfaces and between bony fragment surfaces were defined in the 3D model. Furthermore, bonded contact definitions between cortical and trabecular bone were established. According to previous research, the screws were preloaded with 50 N, and the friction coefficients between bone-to-bone and bone-to-screw were assigned 0.46 and 0.37, respectively [23–26]. Under the isotropic homogeneous linear elastic material model assumptions, material properties for cortical and trabecular bone and titanium alloy cortical screws were allocated independently (Table 2) [27–33].

**Table 2:** Material properties assigned in the FEA set up in accordance with the homogeneous isotropic linear elastic material model

*Material properties*				
Parameters	Unit	Model components		
		Cortical bone	Trabecular bone	Cortical screws (Ti–6Al–4 V)
Modulus of elasticity	(MPa)	19,100	1000.61	115,000
Poisson’s ratio	(−)	0.30	0.30	0.33
Density	(kg m^−3^)	1980	830	4500

*FEA* finite element analysis

### Mesh structure and quality verification

**Fig. 4:**
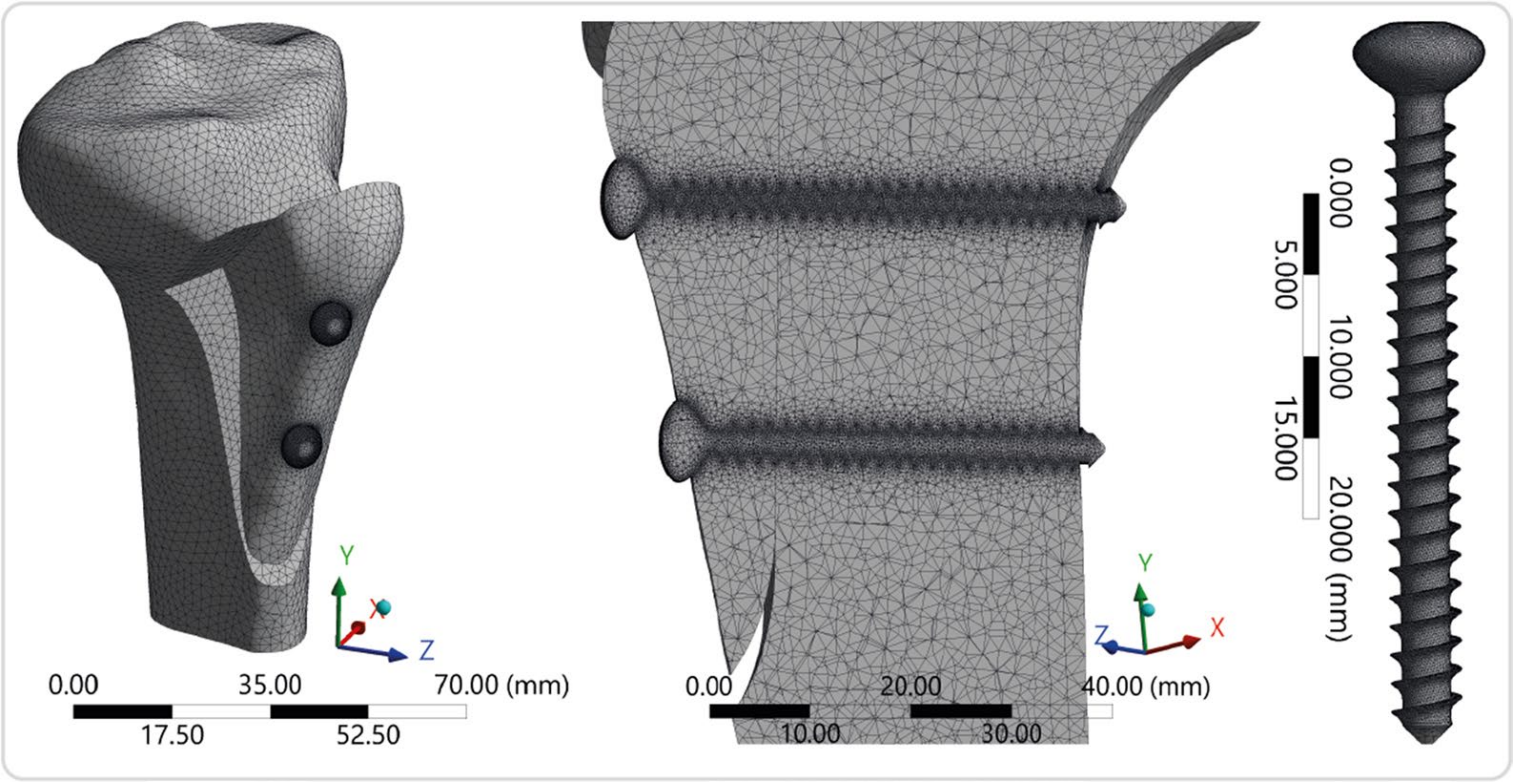
The mesh structure of the model and screws

The accuracy of FEA simulation is considerably affected by the quality of the mesh structure of a model. The skewness metric, which defines how near to ideal a face or cell is in a finite element model, is one of the key quality measurements for a mesh structure in an FEA. The skewness of a distribution may be used to determine its shape and asymmetry, allowing mesh structure verification [34]. A value of zero denotes an equilateral cell (best mesh quality), and a value of one indicates an utterly degenerate cell (worst mesh quality) according to the analysis code’s definition of skewness [35]. As a result, the mesh structure in this study was verified using the skewness measure (mesh quality). The skewness values demonstrated high mesh quality for all of the cases evaluated, with a mean of 0.264 ± 0.01. The final mesh structure of the solid models was created using a curvature-based meshing approach. An average of 1.38 million elements and 2.06 million nodes were found for all solid models. Figure 4 shows a visual representation of the meshing of the models. Each simulation scenario was run separately with identical boundary conditions after completing the preprocessor steps, and then visual and numerical outputs were recorded. A Dell Precision M4800 Series mobile workstation (Intel CoreTM i7 4910MQ CPU @ 2.90 GHz, NVIDIA Quadro K2100M-2 GB, and Physical Memory: 32 GB) was used to solve the problem.

### Assessment of sliding and separation

**Fig. 5:**
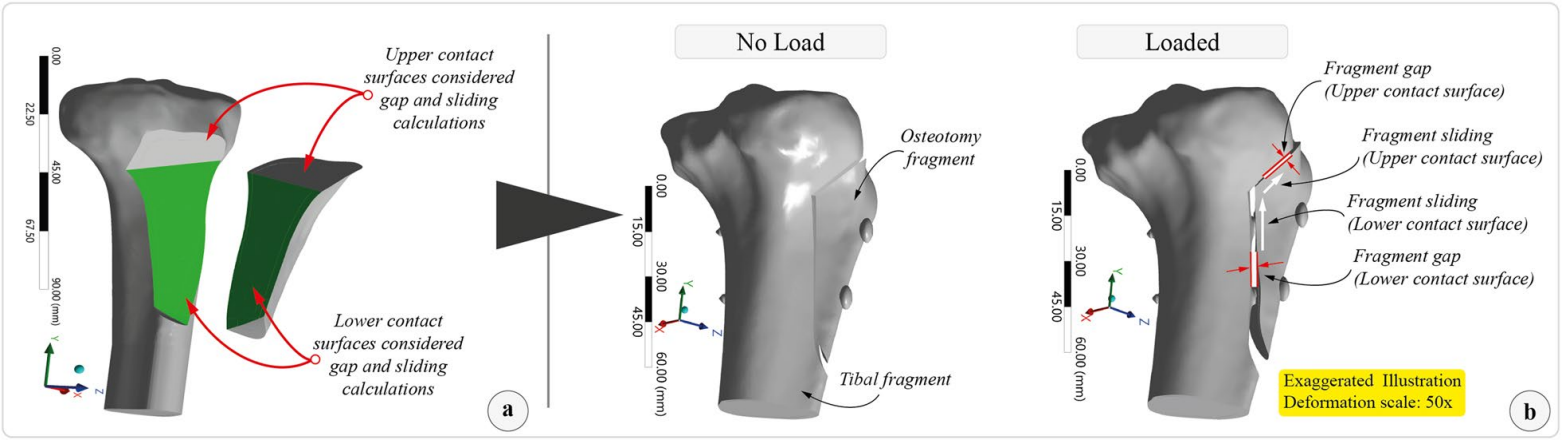
**a** The contact surfaces of the osteotomy (upper and lower surface). **b** The sliding and gap formation was measured after loading independently

The magnitude of maximum gap formation and sliding distance between the osteotomy fragment and the tibia were calculated (Fig. 5). In addition, the equivalent (von Mises) stress and total deformation distributions on the components were retrieved from the simulation results.

## Results

After loading the models with 1654 N patellar tendon traction force, the osteotomy fragment moved superiorly. Since the proximal cut is sloped (bevel-cut osteotomy), the osteotomy fragment slid and rested on the upper tibial surface. Afterward, the upper surface of the osteotomy fragment acted as a fulcrum, and the distal part of the fragment began to separate from the tibia while the screws resisted the displacement.

The average sliding through the upper and lower contact surfaces was the least in the fourth scenario (FEA-001: 0.165 mm, FEA-002: 0.182 mm, FEA-003: 0.133 mm, and FEA-004: 0.128 mm, respectively). The average gap formation at the upper and lower contact surfaces was the least in the third scenario (FEA-001: 1.052 mm, FEA-002: 1.013 mm, FEA-003: 0.996 mm, and FEA-004: 1.025 mm, respectively). The resultant total displacement was the least in the fourth scenario (FEA-001: 0.319 mm, FEA-002: 0.307 mm, FEA-003: 0.333 mm, and FEA-004: 0.245 mm, respectively). Maximum frictional stress and maximum pressure between components on both surfaces were highest in the first scenario (FEA-001). The summary of the results is presented in Table 3.

**Table 3:** The results of four models analyzed in terms of sliding distance, gap formation, resultant displacement, frictional stress, and pressure

FEA output		TTO—FEA-001			TTO—FEA-002		TTO—FEA-003		TTO—FEA-004	
		Upper surface		Lower surface	Upper surface	Lower surface	Upper surface	Lower surface	Upper surface	Lower surface
Maximum sliding distance	(mm)	0.093	0.237		0.124	0.241	0.162	0.104	0.084	0.173
Average sliding	(mm)	0.165			0.182		0.133		0.128	
Maximum gap	(mm)	1.491	0.613		1.470	0.557	1.476	0.516	1.481	0.572
Average gap	(mm)	1.052			1.013		0.996		1.025	
Maximum resultant displacement (entire model)	(mm)	0.319			0.307		0.333		0.245	
Stiffness of the entire model	N/mm	5185			5387		4967		6751	
Maximum frictional stress	(MPa)	25.094	3.802		23.285	3.307	19.287	2.888	21.158	3.255
Maximum pressure	(MPa)	54.552	8.264		50.619	7.188	41.928	6.279	45.996	7.076

*FEA* finite element analysis, *TTO* tibia tubercle osteotomy

**Fig. 6:**
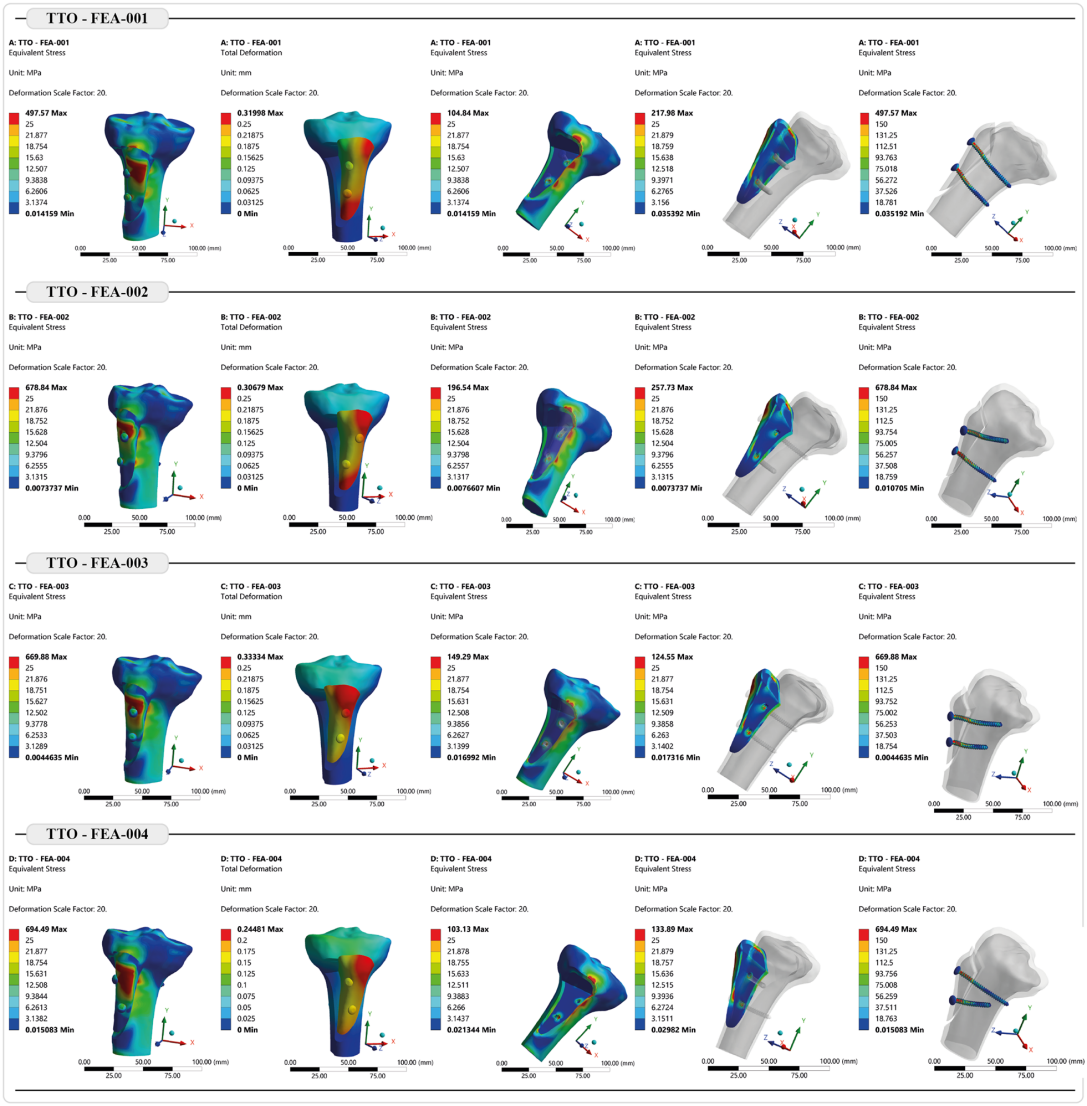
Detailed visual simulation outputs of all tested scenarios

Under predefined loading conditions, no permanent deformation or damage was detected on the screws. However, maximum equivalent (von Mises) stress values on the cortical and trabecular bone around the screw threads and the upper surface fulcrum point locally exceeded their yield stress points reported in previous studies [28, 32, 36, 37]. Figure 6 displays the visual simulation results for each scenario.

## Discussion

The fourth model (FEA-004) demonstrated the least magnitude of sliding distance in the upper surface of the osteotomy as well as the least magnitude of maximum displacement. Therefore, the fourth model emerged as the most favorable screw configuration, with the highest stiffness among these models. Based on the findings of the current study, a divergent screw fixation in which the upper screw is inserted perpendicular to the osteotomy plane and the lower screw is inserted perpendicular to the posterior tibial cortex might be recommended.

The screw configuration proposed in this study has some advantages besides providing better stability. Two previous radiologic studies investigated the safe zone for TTO fixation to prevent iatrogenic popliteal vessel injury, a devastating complication of this procedure [8, 9]. The authors recommended routing the drill and screws toward the posteromedial cortex, which is safe for bicortical fixation. In addition to being a safe zone for screw tips, the posteromedial cortex involves dense bone that provides better screw purchase. However, this analysis could not demonstrate the greatest results for the first model comprising two parallel screws perpendicular to the osteotomy plane. On the other hand, the popliteal artery gives its major peripheric branches approximately 5 cm distal to the tibial joint line. Thus, the risk of injury is higher in the upper screw than in the lower screw [38]. The divergent screw configuration proposed in this study might also significantly reduce the risk of vascular injury without impairing stability.

Secondly, this screw configuration might reduce the number of implant removal operations. The tibial tubercle is in contact with various surfaces during kneeling. Due to its prominence and relatively thin overlying soft tissue, the skin over the tibial tubercle area is vulnerable to irritation. Moreover, prominent hardware causes significant discomfort. Therefore, hardware removal is the most frequent secondary surgical intervention performed following Fulkerson osteotomy [5]. A previous systematic review including 1055 TTO procedures reported an overall incidence of 19% hardware removal [39]. In another systematic review, 49% of the cases had undergone implant removal following Fulkerson osteotomy [3]. Moreover, some authors have recommended routine removal of the hardware after the union of TTO, regardless of the presence and severity of the symptoms [40, 41]. Since screw placement perpendicular to Fulkerson osteotomy allows better positioning of the screw head, deep to the tibialis anterior muscle belly, as shown in Fig. 1, this configuration seems more feasible to overcome the potential skin irritation. The findings of this study also favor the perpendicular insertion of the upper screw for better stability. Screws inserted perpendicular to the osteotomy plane from lateral to medial direction do not adversely affect the stability. The screw heads move away from the anterior direct contact surface and are covered by the muscle mass.

The majority of previous biomechanical studies have assessed the fixation techniques of TTO used in the extended exposure of revision knee arthroplasty [6, 11–19, 42]. Only a few studies have focused on the biomechanical features of flat and oblique TTO used to treat PF instability. These two osteotomies are technically different from each other. While medial displacement is not performed in TT osteotomy used in revision knee arthroplasty, medialization is performed in PF instability surgery. Secondly, the plane of osteotomy is oblique. Therefore, although these two osteotomies are similar in three-dimensional shape, the contact surface area decreases in medialization, and the patellar tendon loading condition is changed, so both osteotomies should be evaluated differently.

Cosgarea et al. [11] compared the fixation strengths of two 3.5 mm bicortical screws in flat and oblique TTOs in cadaveric tibiae. The authors reported superior results for flat osteotomy, despite a greater contact area in the oblique osteotomy line. However, flat osteotomies, also known as Elmslie–Trillat, are no longer preferred because they increase patellofemoral contact pressures and cause osteoarthritis in the long term. Another cadaveric study, conducted by Warner et al. [6], compared two 4.5 mm and three 3.5 mm cortical screws in Fulkerson osteotomy. Their results indicated more robust stability in two 4.5 mm screw fixations than in three 3.5 mm screw fixations. Using a cadaver model, Nurmi et al. [14] compared the bioabsorbable 4.5 mm poly-l-lactide (PLLA) screws with stainless-steel screws in the flat osteotomy. Although the stainless-steel screws demonstrated approximately two-fold higher stiffness, the authors reported that the PLLA screws could resist the tractive forces of active knee extension. Furthermore, Ünal et al. [43] have reported favorable clinical outcomes in ten patients who had undergone Fulkerson osteotomy fixed with 4.8-mm headless bioabsorbable screws made of magnesium. Therefore, using bioabsorbable screws in the fixation of TTO seems practicable since it may avoid secondary procedures due to common hardware-related skin irritation.

Three previous FEA studies investigated the fixation of TTO used in the extended exposure of knee arthroplasty without medialization [15–17]. The first study assessed the fixation of flat tubercle osteotomy with two 4.5 mm titanium screws implanted in the configurations as follows: parallel horizontal, parallel downward, parallel upward, trapezoid, and divergent [16]. The authors reported the highest stability for parallel downward screw configuration depending on the least magnitude of fragment displacement and gap formation. The second study assessed three configurations of flat tubercle osteotomy (step cut, bevel cut, and straight cut) and various fixation configurations with two 4.5 mm screws [15]. Step cut was reported to be the most stable osteotomy configuration, based on the buttressing effect inherent in the step-cut technique. The third FEA study investigated the effect of gap formation between the distal and proximal sides of the tibial tubercle fragment on the stability of the fixation with different screw configurations[17]. The proximal gap model resulted in inferior results compared to the distal gap model in all screw configurations. Among the screw configurations, two parallel downward screws resulted in the highest stability in both models. Since the effect of tubercle medialization or anteromedialization has not been studied in the studies mentioned above [15–17], the present study is more predictive for TTO used to treat PF instability. Unlike these two studies, including the screw configurations with alterations in the sagittal plane, the present research biomechanically compared the screw configurations with modifications in the axial plane to avoid prominent hardware as much as possible.

Patellar height can be normalized by distalizing the TT during Fulkerson osteotomy in patients with accompanying patella alta. In the last FEA study, the biomechanical stability of the various 3.5 mm, 4.5 mm cortical screws and 3.5 mm 1/3 tubular plate-screw augmentation options were compared in Fulkerson osteotomy with distalization procedure [18]. Since the distal hinge and the proximal contact is lost in distalization procedures, the osteotomy becomes inherently unstable compared to a standard Fulkerson osteotomy. Thus, the authors recommended plate-screw-augmented fixation to achieve maximum stability. Although the osteotomy modeling differed from the current study, the screw configuration was similar, and they advocated inserting the screws perpendicular to the osteotomy plane. All these previous FEA studies show that changing the screw configuration might increase the stability of the fixation construct in TT osteotomies.

The current study has various limitations as well as strengths. The inherent constraints of FEA must be considered while evaluating the results. Several preset assumptions were used, such as boundary conditions and material properties. Secondly, since the data in this FEA study are absolute values, a statistical comparison was not made. Despite the limitations of this study, it also has some strengths. CT data of a patient with patellofemoral instability and an increased TT–TG distance were employed to create a precise model to simulate the clinical reality. Since the bone is not uniform, the cortical and trabecular bones were separately defined with a bonded contact model. Although a cadaveric experiment did not validate the results of this FEA, it provides important information about the biomechanical characteristics of Fulkerson osteotomy and its fixation.

## Conclusion

A divergent screw configuration in which the upper screw is inserted perpendicular to the osteotomy plane and the lower screw inserted perpendicular to the posterior tibial cortex (FEA-004) might be a better option for the fixation of Fulkerson osteotomy. Surgeons who prefer two 4.5 mm cortical screws for Fulkerson osteotomy fixation can achieve a more stable fixation by applying this simple modification in screw configuration. In addition, this modification may reduce the risk of neurovascular injuries and thus allow safer bicortical screw fixation. Finally, it can reduce the need for implant removal by shifting the screw heads away from the direct contact area of the knee. However, these propositions need to be supported by further clinical studies.

## Data Availability

The datasets generated during and/or analyzed during the current study are available from the corresponding author upon reasonable request.

## Abbreviations

AO: Arbeitsgemeinschaft für Osteosynthesefragen
CT: Computerized tomography
FEA: Finite element analysis
PF: Patellofemoral
Ti: Titanium
TT: Tibial tubercle
TTO: Tibial tubercle osteotomy
TT–TG: Tibial tubercle–trochlear groove
Q angle: Quadriceps angle
3D: Three-dimensional
